# Pivotal role of CD103 in the development of psoriasiform dermatitis

**DOI:** 10.1038/s41598-020-65355-9

**Published:** 2020-05-20

**Authors:** Takehito Fukui, Tomohiro Fukaya, Tomofumi Uto, Hideaki Takagi, Junta Nasu, Noriaki Miyanaga, Yotaro Nishikawa, Haruhiko Koseki, Narantsog Choijookhuu, Yoshitaka Hishikawa, Yoshihiro Yamashita, Katsuaki Sato

**Affiliations:** 1 Division of Immunology, Department of Infectious Diseases, Faculty of Medicine, University of Miyazaki, 5200 Kihara, Kiyotake Miyazaki, 889-1692 Japan; 2 Department of Oral and Maxillofacial Surgery, Faculty of Medicine, University of Miyazaki, 5200 Kihara, Kiyotake Miyazaki, 889-1692 Japan; 3 Department of Otolaryngology, Head and Neck Surgery, Faculty of Medicine,University of Miyazaki, 5200 Kihara, Kiyotake Miyazaki, 889-1692 Japan; 4 Department of Dermatology, Faculty of Medicine, University of Miyazaki, 5200 Kihara, Kiyotake Miyazaki, 889-1692 Japan; 5 Laboratory for Developmental Genetics, RIKEN Center for Integrative Medical Sciences, 1-7-22 Suehiro-cho, Tsurumi-ku, Yokohama, Kanagawa 230-0045 Japan; 6 Division of Histochemistry and Cell Biology, Department of Anatomy, Faculty of Medicine, University of Miyazaki, Miyazaki, 889-1692 Japan; 7 Japan Agency for Medical Research and Development (AMED), 1-7-1 Otemachi, Chiyoda-ku, Tokyo 100-0004 Japan

**Keywords:** Autoimmunity, Psoriasis

## Abstract

The integrin αE known as CD103 binds integrin β7 to form the complete heterodimeric integrin molecule αEβ7. CD103 is mainly expressed by lymphocytes within epithelial tissues of intestine, lung, and skin as well as subsets of mucosal and dermal conventional dendritic cells (cDCs). CD103 has been originally implicated in the attachment of lymphocytes to epithelium in the gut and skin through the interaction with E-cadherin expressed on intestinal epithelial cells, keratinocytes, and Langerhans cells (LCs). However, an impact of CD103 on the cutaneous immune responses and the development of inflammatory skin diseases remains elusive. Here, we report that CD103 regulates the development of psoriasiform dermatitis through the control of the function of cDCs. Deficiency in CD103 exacerbates psoriasiform dermatitis, accompanied by excessive epidermal hyperplasia and infiltration of inflammatory leukocytes. Furthermore, deficiency in CD103 not only accelerates the production of proinflammatory cytokines in psoriatic lesions but also promotes the generation of lymphocytes producing interleukin (IL)-17 in the skin-draining peripheral lymph nodes (PLNs). Under the deficiency in CD103, cDCs localized in PLNs enhance cytokine production following activation. Thus, our findings reveal a pivotal role for CD103 in the control of the function of cDCs to regulate cutaneous inflammation in psoriasiform dermatitis.

## Introduction

Dendritic cells (DCs) are essential antigen (Ag)-presenting cells (APCs) to produce multiple cytokines and activate naïve T cells during primary responses upon recognition of the pathogens through pattern recognition receptors (PRRs)^1–3^. Therefore, DCs play critical roles in orchestrating the immune system, linking innate and adaptive immunity^4–6^. DCs are classified into two major subsets, classical or conventional DCs (cDCs) and plasmacytoid DCs (pDCs)^1–3^. cDCs display an unique ability to prime naïve T cells to induce various types of effector T (T_eff_) cells due to the remarkable expressions of major histocompatibility complex (MHC) molecules and costimulatory molecules^1–3^. Thus, cDCs has been recognized as central to regulate the direction of adaptive immune responses for the protection against microbial infections. On the other hand, pDCs are characterized to secrete the massive amounts of type I IFN (IFN-I) following recognition of viral nucleic acids (NAs) through endosomal TLR7/9^4^. Therefore, pDCs have been considered as critical immune cells of antiviral responses.

Psoriasis, one of the most common chronic inflammatory cutaneous disease affecting around 2% of the global population, is characterized by several pathological features including red, scaly, raised plaques at different body sites, finally causing the impairment of the skin barrier function^5–8^. Histological manifestations of psoriasis include diffuse epidermal hyperplasia (acanthosis) owing to hyperproliferation of keratinocytes and parakeratosis that is caused by an aberrant differentiation of keratinocytes^8^, as well as prominent inflammatory infiltrating leukocytes consisting of T cells, macrophages, and DCs in the dermis and neutrophils in the epidermis^9–11^. Whereas several inflammatory cytokines such as interleukin (IL)−1β, IL-6, and tumor necrosis factor (TNF)-α, and type I interferon (IFN-I) have been associated with the priming and skewing of psoriatic inflammation^11,12^, recent studies have shown that the axis of IL-22-IL-23-IL-17 cytokine is critical for the development of skin immunopathology^13–16^. While the exact initiation process for psoriasis is not fully understood, IL-23 secreted by macrophages and cDCs induces the expansion and activation of T helper 17 (T_H_17) cells, T cytotoxic 17 (T_C_17) cells, and γδT cells, as well as type 3 innate lymphoid cells (ILC3) to produce IL-17 and their related cytokines in the process of psoriatic skin inflammation^9,13–16^.

The integrin αE (ITGAE) known as CD103 binds integrin β7 (ITGB7) to form the complete heterodimeric integrin molecule αEβ7^17–19^. CD103 is primarily expressed by epithelial T cells, including CD4^+^ and CD8^+^ T cells and γδTCR^+^ T cells^17–20^, as well as other leukocytes such as a population of cDCs^1–3^ in the intestine, lung, and skin. CD103 has been originally implicated in the attachment of lymphocytes to epithelial tissues in the gut and skin through the interaction with E-cadherin expressed on intestinal epithelial cells, keratinocytes, and Langerhans cells (LCs)^17–20^. In several experimental models with CD103-deficient mice, CD103 has been required for the localization of CD8α^+^ intraepithelial lymphocytes (IELs) bearing αβT-cell receptor (TCR) in the gut^21^ and epidermal γδTCR^+^ T cells in the skin^22^. CD103 has been involved in the destruction of pancreatic islet allografts^23^ and intestines in graft-versus-host diseases (GVHD)^24^ through the promotion of the migration of the pathogenic CD8^+^ T cells into epithelial compartments. Furthermore, it has been shown that CD103 contributes to the development of airway inflammation in asthma^25^. On the other hand, CD103 is dispensable for the control of a chronic antiviral immune response^26^ and intestinal immunity to helminth infection^27^. In the skin pathogenesis, CD103 has reportedly participates in the development of allergic contact hypersensitivity through the regulation of the retention of CD4^+^Foxp3^+^ regulatory T (T_reg_) cells to the inflamed skin^28^ as well as the induction of cutaneous inflammatory disorder, that was affected by some environmental conditions and/or in the context of other genetic factors^20^. While CD103 has reportedly no obvious pathogenic role in psoriasiform skin lesions of transgenic (Tg) mice overexpressing human transforming growth factor (TGF)-β1 under the regulation of the keratin 5 promoter within the epidermis (K5.hTGFβ1 Tg mice), which displayed a spontaneous development of psoriatic dermatitis^29^, the potential role of CD103 in the control of the pathogenesis of psoriasiform dermatitis and other cutaneous inflammatory disorders remains elusive.

In this study, we show that impact of CD103 on the development of psoriasiform dermatitis mediated through the control of the function of cDCs in the skin-draining peripheral lymph nodes (PLNs) with use of CD103-deficient mice.

## Results

### Deficiency of CD103 aggravates skin inflammation

**Figure 1:**
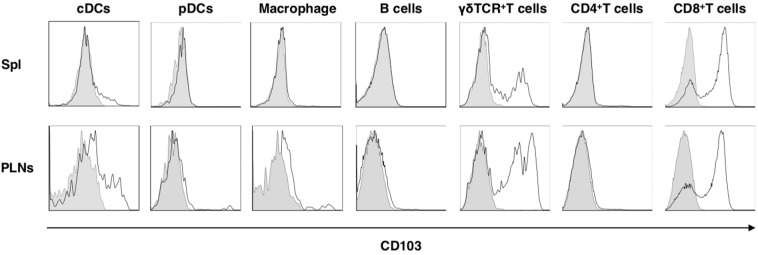
The expression of CD103 on leukocytes in lymphoid tissues. The expression of CD103 on leukocytes derived from WT mice and *Cd103*^−/−^ mice. Data are presented by a histogram, in which open or gray indicate WT mice or *Cd103*^−/−^ mice, respectively. The results are representative of at least three independent experiments.

**Figure 2:**
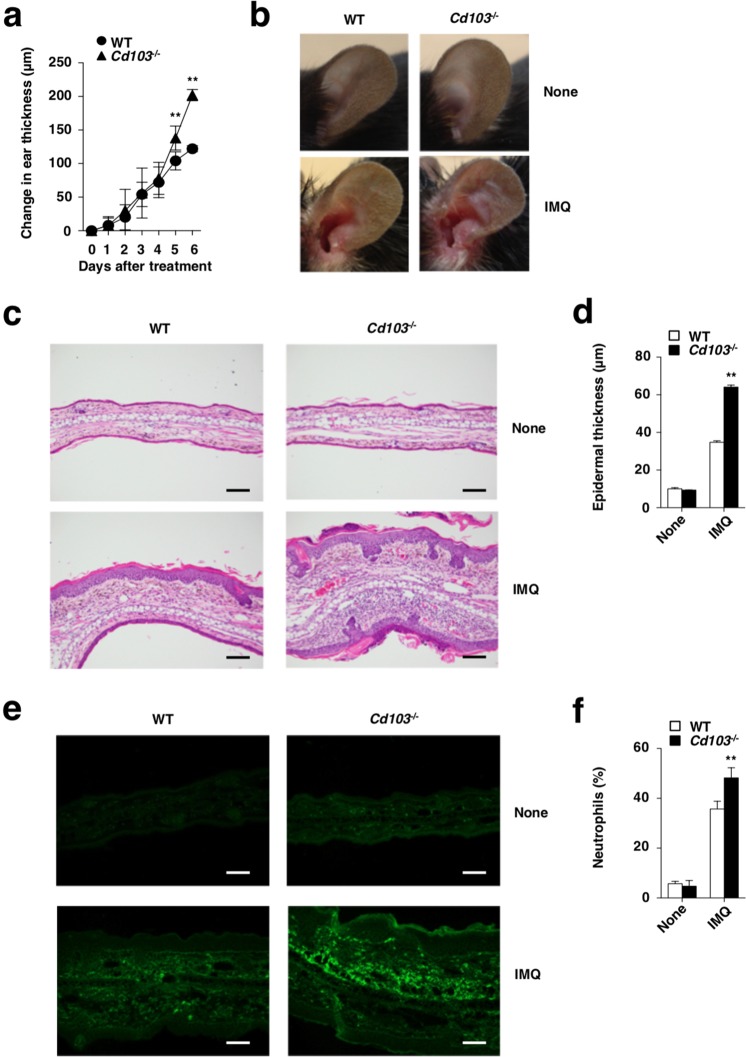
CD103 deficiency exacerbates psoriatic dermatitis. WT mice (n = 5) and *Cd103*^−/−^ mice (n = 5) received topical treatment with IMQ on the left ear skin every day for 6 days. (**a**) Ear thickness was evaluated for 6 days. Data were representative of five individual samples in a single experiment. **P < 0.01 compared with WT mice. (**b**) Representative pictures of ear skin lesions at 6 days. (**c,d**) Hematoxylin and eosin (H&E) sections (magnification; 20x) of ear skin at days 0 (None) and 6 (IMQ) (**c**), and epidermal thickness was evaluated at days 0 (None) and 6 (IMQ) (**d**). Data were representative of five individual samples in a single experiment. **P < 0.01 compared with WT mice. (**e**) Immunohistochemical sections (magnification; 20x) for detecting Gr-1 of ear skin at days 0 (None) and 6 (IMQ). (**f**) The frequency of neutrophils in ear skin at days 0(None) and 6 (IMQ). Data are the mean ± s.d. in four individual samples in a single experiment. **P < 0.01 compared with WT mice. The results are representative of at least three independent experiments with similar results.

To address the role of CD103 *in vivo*, we created mice lacking the *Cd103* gene (*Cd103*^−/−^) (Supplementary Fig. 1). *Cd103*^−/−^ mice were born at the expected Mendelian frequencies and were apparently healthy. Whereas both wild-type (WT) mice and *Cd103*^−/−^ mice exhibited similar cellularity in skin-draining lymph nodes (PLNs), *Cd103*^−/−^ mice exhibied the lower frequencies of cDCs and macrophages, but not other leukocytes, in spleen (Spl) than WT mice (Supplementary Figs. 2, 3). While several types of leukocytes displayed various expression level of CD103 on the cell surface in skin-draining PLNs and Spl in WT mice, their expressions were absence in *Cd103*^−/−^ mice (Fig. 1). Upon topical application of imiquimod (IMQ), a synthetic Toll-like receptor 7 (TLR7) ligand, on the ear skin^11,13^, *Cd103*^−/−^ mice displayed a more prominent psoriasiform dermatitis, such as thickening and scaling than WT mice (Fig. 2a,b). Histological analyses revealed that *Cd103*^−/−^ mice showed a more significant parakeratosis, acanthosis, and Munro’s microabscessess than WT mice after topical application of IMQ (Fig. 2c,d). Furthermore, *Cd103*^−/−^ mice exhibited the prominent skin infiltration of mononuclear cells, including Gr-1^+^ granulocytes in cutaneous inflammatory tissues when compared with WT mice upon topical application of IMQ (Fig. 2c,e,f).

Collectively, these results indicate that an absent of CD103 exacerbates psoriasiform dermatitis.

### Deficiency of CD103 accelerates skin inflammation

**Figure 3:**
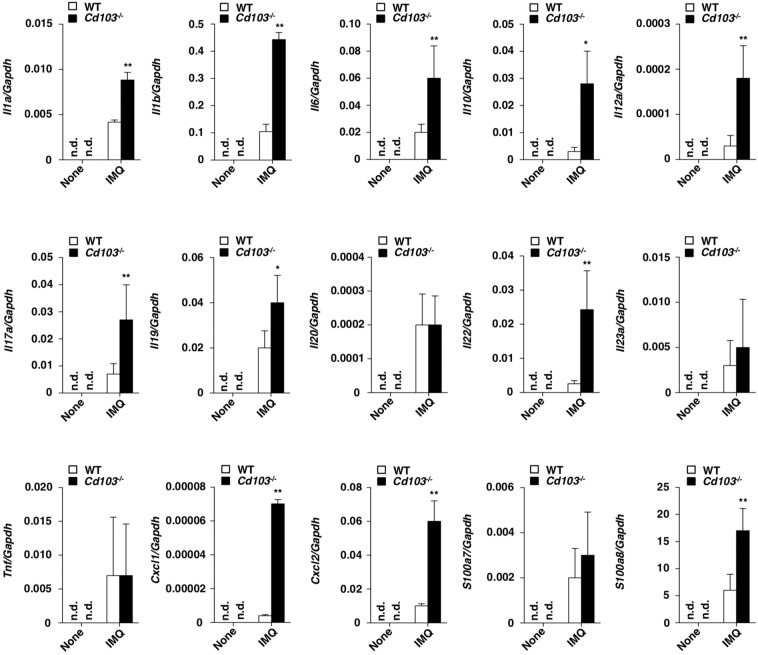
CD103 deficiency enhances the psoriatic inflammation. Transcriptional expressions of cytokines, chemokines, and epithelial inflammation-related molecules in ear skin at days 0 and 6 after topical application of IMQ on the ear skin in WT mice (n = 3) and *Cd103*^−/−^ mice (n = 3), and the expression was normalized to the *Gapdh* transcript. Data are the mean ± s.d. in three to five individual samples in a single experiment. *P < 0.05, **P < 0.01 compared with WT mice. The results are representative of at least three independent experiments.

To clarify the role of CD103 in the regulation of psoriasiform inflammation, we examined the transcriptional expressions of cytokines and chemokines, as well as epithelial inflammation-related molecules, in psoriatic lesions in WT mice and *Cd103*^−/−^ mice (Fig. 3). After topical application of IMQ, *Cd103*^−/−^ mice exhibited higher transcriptional expressions of *Il1a Il1b*, *Il6*, *Il10*, *Il12a*, *Il17a*, *Il19*, *Il22*, *Cxcl1*, *Cxcl2*, and *S100a8* than WT mice.

**Figure 4:**
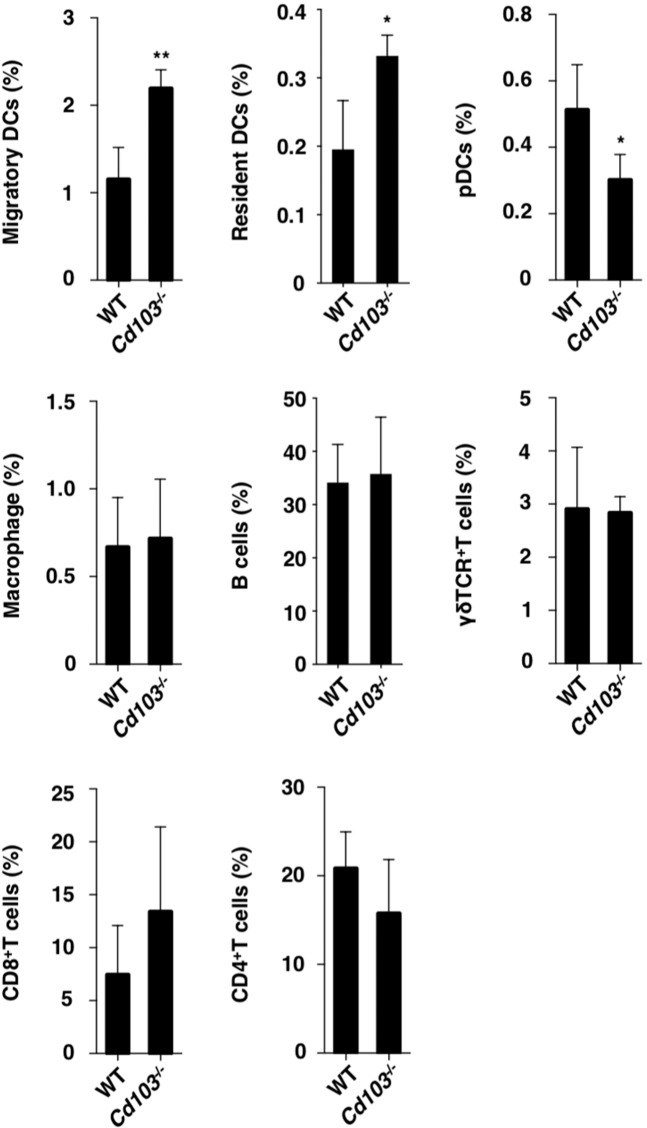
Absence of CD103 promotes the accumulation of inflammatory leukocytes in skin-draining PLNs. The frequency of leukocytes in the skin-draining PLNs at day 6 after topical application of IMQ on the left ear skin every day for 6 days in WT mice (n = 5) and *Cd103*^−/−^ mice (n = 5). Data are the mean ± s.d. in three to five individual samples in a single experiment. *P < 0.05, **P < 0.01 compared with WT mice. The results are representative of at least three independent experiments.

We also examined the impact of CD103 on the constituency of inflammatory leukocytes in the skin-draining PLNs after the initiation of psoriasiform dermatitis. In the skin-draining PLNs, *Cd103*^−/−^ mice displayed higher or lower accumulation of migratory MHC II^hi^CD11c^med^ cDCs (migratory cDCs) and resident MHC II^med^CD11c^hi^ cDCs (resident cDCs), or pDCs than WT mice (Fig. 4 and Supplementary Fig. 4).

Collectively, these results indicate that a deficiency of CD103 enhances the formation of the pathogenic cytokine and leukocyte milieu to induce psoriatic inflammation.

### Deficiency of CD103 enhances the activation status of cDCs

**Figure 5:**
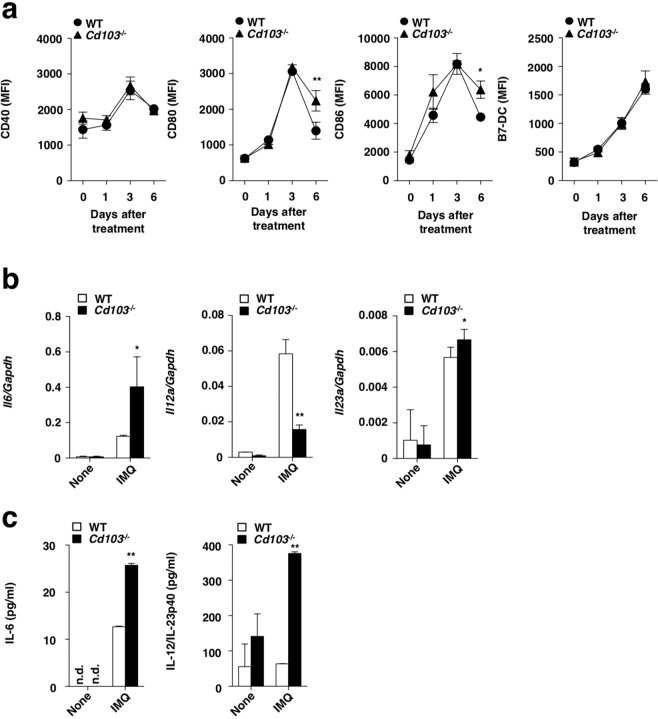
CD103 deficiency enhances the activation of cDCs in response to TLR7 ligand. (**a**) WT mice and *Cd103*^−/−^ mice received topical treatment with or without IMQ on the left ear skin every day for 6 days. The expressions of costimulatory molecules on cDCs before and the indicated days after topical application of IMQ. Data are the mean fluorescence intensity (MFI) ± s.d. in three individual samples in a single experiment. *P < 0.05 compared with WT mice. (**b,c**) cDCs were derived from the skin-draining PLNs in WT mice and *Cd103*^−/−^ mice were not stimulated (None) or stimulated with IMQ (IMQ). The transcriptional expressions (**b**) and the production (**c**) of cytokines in cDCs. Data are the mean ± s.d. in three individual samples in a single experiment. *P < 0.05, **P < 0.01 compared with WT mice. The results are representative of at least three independent experiments.

To address the influence of the absence of CD103 on the activation of cDCs, we examined the surface expressions of costimulatory molecules on cDCs in the skin-draining PLNs in psoriatic dermatitis. Upon topical treatment of IMQ on day 6, cDCs from *Cd103*^−/−^ mice displayed marked expression levels of CD80 and CD86 as compared with those from WT mice (Fig. 5a).

We further compared the capacity of cDCs between WT mice and *Cd103*^−/−^ mice following stimulation with IMQ. cDCs derived from skin-draining PLNs in *Cd103*^−/−^ mice exhibited higher expressions of *Il6*, *Il23a*, and lower expressions of *Il12a* than those in WT mice (Fig. 5b). Similar tendency was observed in splenic cDCs in WT mice and *Cd103*^−/−^ mice (Supplementary Fig. 5a). Furthermore, cDCs derived from skin-draining PLNs in *Cd103*^−/−^ mice exhibited higher productions of IL-6 and IL-12/IL-23p40 than those in WT mice (Fig. 5c). On the other hand, cDCs derived from skin-draining PLNs in *Cd103*^−/−^ mice displayed the enhanced ability to generate CD8^+^ T cells producing IL-17 (T_C_17 cells) and the reduced ability of IFN-γ-producing CD8^+^ T cells (T_C_1 cells) than those in WT mice (Supplementary Fig. 5b).

Taken together, these results indicate that the absence of CD103 enhances the activation status of cDCs to generate T_C_17 cells.

### Deficiency of CD103 promotes the differentiation of T_C_17 cells for the progression of psoriatic inflammation

**Figure 6:**
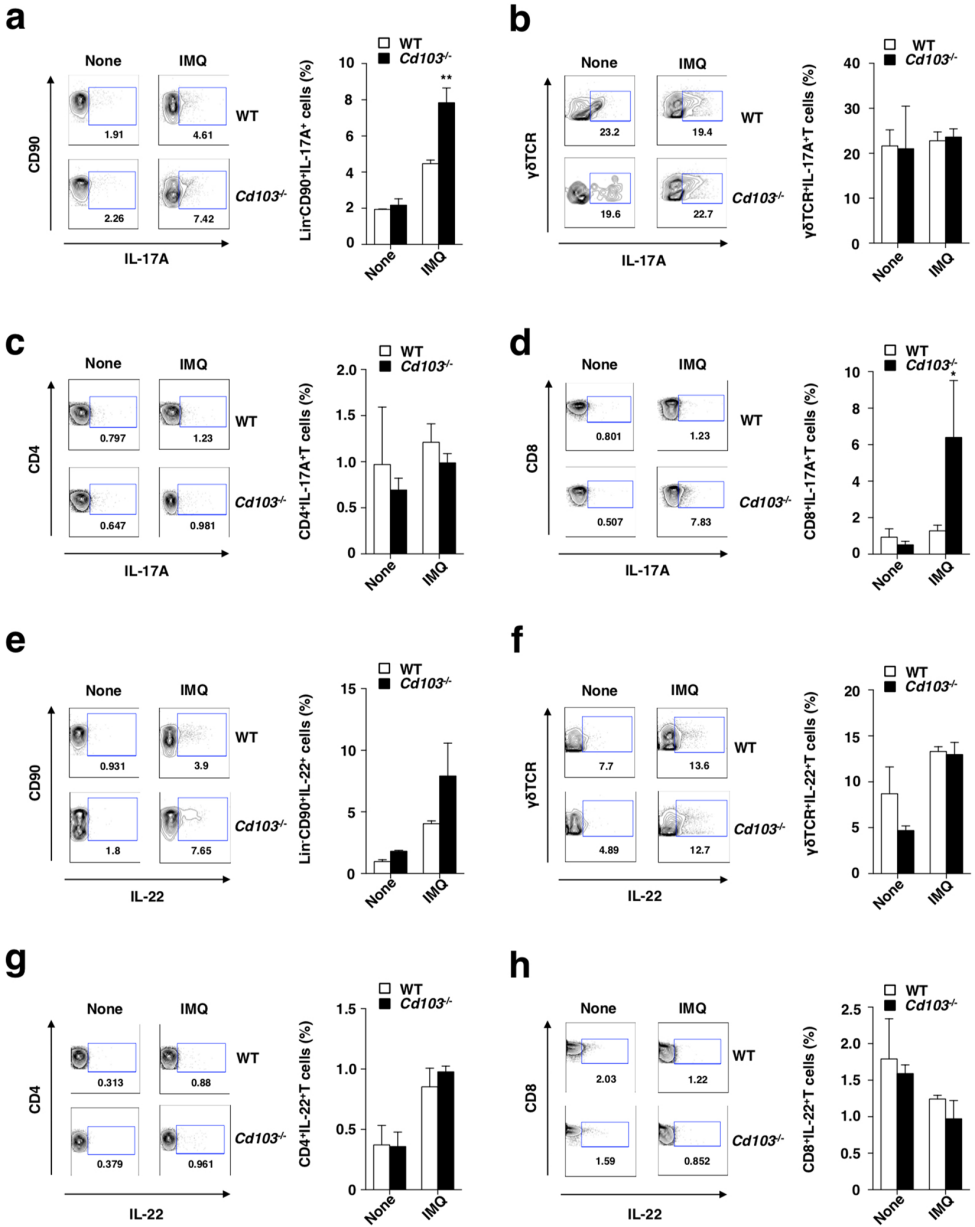
CD103 deficiency enhances the generation of IL-17A-prodcing lymphocytes in the skin-draining PLNs in the progression of psoriasiform skin inflammation. WT mice (n = 3) and *Cd103*^−/−^ mice (n = 3) were received topical treatment with IMQ on the left ear skin every day for 6 days. The frequencies of IL-17A-producing cells (**a–d**) and IL-22-producing cells (**e–h**) among innate lymphocytes (**a,e**), γδTCR^+^ T cells (**b,f**), CD4^+^ T cells (**c,g**), and CD8^+^ T cells (**d,h**) in the skin-draining PLNs at days 0 and 6. Data are presented as a contour plot, and numbers mean the proportion of the indicated cell populations in each gate (left panel). Data are the mean ± s.d. in three individual samples in a single experiment (right panel). *P < 0.05, **P < 0.01compared with WT mice. The results are representative of at least three independent experiments.

To assess the impact of CD103 on the regulation of the axis of IL-22-IL-23-IL-17 cytokine, we compared the induction of IL-17A- and IL-22-producing lymphocytes in the skin-draining PLNs between WT mice and *Cd103*^−/−^ mice following initiation of psoriasiform dermatitis. The psoriatic *Cd103*^−/−^ mice displayed higher accumulation of IL-17A-producing ILCs and T_C_17 cells than the psoriatic WT mice (Fig. 6a,d, Supplementary Figs. 6a,b, and 7a,d), whereas their accumulation of other IL-17A-producing lymphocytes, such as γδTCR^+^ T cells and T_H_17 cells, were comparable (Fig. 6b,c and Supplementary Fig. 7b,c). On the other hand, there was no obvious difference in the proportions of IL-22-producing lymphocytes (Fig. 6e–h and Supplementary Fig. 7e–h). We also observed that the accumulation of IL-17A- or IL-22-producing lymphocytes in the psoriatic dermal tissues was higher in *Cd103*^−/−^ mice than WT mice (Supplementary Fig. 8).

We further examined the influence of the absence of CD103 on the capacity of CD8^+^ T cells to differentiate into T_C_17 cells. CD8^+^ T cells derived from *Cd103*^−/−^ mice displayed an enhanced ability to develop into T_C_17 cells rather than T_C_1 cells as compared with those derived from WT mice (Supplementary Fig. 9).

Taken together, these results indicate that the deficiency of CD103 enhances the generation of IL-17A-producing lymphocytes to amplify the psoriatic inflammation.

## Discussion

Although the accumulating evidences suggest that CD103 is involved in the pathogenesis of some immune-mediated diseases such as GVHD, asthma, and allergic contact hypersensitivity, how CD103 controls immune responses leading to the development of cutaneous inflammatory disorders remains unclear. In this study, we revealed that CD103 not only inhibits the function of cDCs and but also impairs the intrinsic potential of CD8^+^ T cells to differentiate into T_C_17 cells to form the pathogenic cytokine and leukocyte milieu for the development of psoriasiform dermatitis.

It has been reported that CD103 is required for the localization of some lymphocytes, including CD8α^+^ IELs and epidermal γδTCR^+^ T cells, to epithelial tissues in the gut^21^ and skin^22^. Furthermore, *Cd103*^−/−^ mice on the 129/SV × BALB/c background reportedly developed the cutaneous inflammatory disorder^20^. On the other hand, we observed that *Cd103*^−/−^ mice on the C57BL/6 background exhibited normal cellularity in skin-associated lymphoid tissues, and they did not develop the spontaneous inflammatory skin disorders under our experimental conditions. Therefore, CD103 is dispensable for the retention of lymphocytes to the skin-associated lymphoid tissues and the regulation of spontaneous inflammatory responses on the C57BL/6 background. These discrepancies might explain that CD103 controls the function of immune cells related to the immunopathogenesis depending on the context of other genetic factors and/or the environmental conditions.

While CD103 is primarily expressed by epithelial leukocytes, the potential role of CD103 in the development of psoriasiform dermatitis remains to be determined. We showed that the deficiency of CD103 aggravated the epithelial pathogenesis and the cutaneous infiltration of mononuclear cells in IMQ-induced psoriasiform dermatitis. Collectively, these results suggest that CD103 regulates the function of the inflammatory leukocytes to suppress the development of psoriasiform dermatitis. On the other hand, previous studies have shown that the deficiency of CD103 did not exacerbate skin pathogenesis in K5.hTGFβ1 Tg mice. Although the reason why the roles of CD103 in the development of psoriasiform dermatitis differs in these models remain unclear, the discrepancies might be due to their distinct mechanisms underlying the initiation and progression of skin inflammation.

Although the several axes of immune dysregulation are supposed to link to the pathogenesis of psoriasiform dermatitis, how CD103 controls the immune responses leading to the cutaneous inflammation remains unclear. Our results showed that CD103 deficiency led to the enhanced productions of various inflammatory cytokines, chemokines, and antimicrobial peptides in the pathogenic skin lesions. Taken together, these results suggest that CD103 inhibits the psoriasiform skin inflammation through the suppression of the formation of the pathogenic cytokine milieu.

Although several subsets of cDCs reportedly express CD103, the role of CD103 in the control of their function remains unclear. We showed that the deficiency of CD103 enhanced the accumulation of migratory cDCs and resident cDCs, but not other leukocytes, in the skin-draining PLNs in the development of psoriasiform dermatitis. Furthermore, the deficiency of CD103 not only enhanced the expression levels of costimulatory molecules but also augmented the secretion of IL-23. Concomitantly, the absence of CD103 enhanced the ability of cDCs to generate T_C_17 cells under T_C_17-polarized condition. Taken together, these results suggest that CD103 regulates the function of cDCs for the IL-23-mediated generation of Tc17 cells to inhibit the progression of psoriasiform dermatitis.

Whereas the IL-17A/IL-22-producing lymphocytes, known as the downstream effector cells of IL-23-IL-17 cytokine axis, are essential for the development of psoriasis^14^, how CD103 regulates the generation and the function of these pathogenic lymphocytes remains unclear. Our results showed that the deficiency of CD103 enhanced the generation of IL-17A-producing ILCs and T_C_17 cells in the skin-draining PLNs. On the other hand, the absence of CD103 promoted the development of CD8^+^ T cells into T_C_17 cells under Tc17-polarized culture condition, indicating that CD103 provide the intrinsic control of the differentiation of these pathogenic CD8^+^ T cells. Collectively, these results suggest that CD103 not only act on cDCs to inhibit the establishment of the milieu of IL-23-IL-17 cytokine axis but also directly impairs the ability of CD8^+^ T cells to differentiate into Tc17 to ameliorate the development of psoriasiform dermatitis.

In the present study, we used *Cd103*^−/−^ mice to clarify the intrinsic role of CD103 in the development of psoriasiform dermatitis, and analysis of this strain revealed the influence of its deficiency on the functions of cDCs and CD8^+^ T cells during the disease progression. Further studies will be needed to determine the individual impact of CD103 on their function *in vivo* for the control of development of psoriasiform dermatitis by creating *Cd103*^flox/flox^ mice to analyze cDC- or CD8^+^ T cell-specific CD103-ablation in mice.

In conclusion, we described that CD103 constitutes critical negative regulatory functions in cDCs and CD8^+^ T cells, it which it prevents the formation of the milieu of the pathogenic cytokine and inflammatory leukocytes for the inhibition of the development of psoriasiform dermatitis. Thus, a better understanding of precise mechanisms how CD103 regulates their functions might open new avenues for providing a new therapy for skin inflammatory immune disorders.

## Methods

### Mice

As described previously^4,11,16^, the following 6- to 12-week-old mice were used in this study. C57BL/6 mice (Japan Clea) and B6.*Cd103*^−/−^ mice as described below. All mice were bred and maintained in specific pathogen-free (SPF) conditions in the animal facility at the University of Miyazaki. All experiments were performed in accordance with institutional guidelines and approved by the Animal Experiment Committee (No. 2019-501, No. 2019-502, and No. 2019-506) and Gene Recombination Experiment Committee (No. 569).

### **Generation of*Cd103***^**−/−**^**mice**

The generation of mice was performed according to the precious reports^4,16^. The targeting vector for *Cd103*^−/−^ mice was constructed in the pBluescript vector by using a 2.0-kilobase (kb) genomic fragment (left arm) upstream of *Cd103* exon 1, and a 4.0-kb genomic fragment (right arm) downstream of exon 1 cloned from a modified bacterial artificial chromosome (BAC) clone, RP23-263M10 (Children’s Hospital Oakland Research Institute), containing the complete *Cd103* gene. The left and right arm were custom-made using GeneArt® (Life Technologies), and each of the 5′- and 3′-ends was tagged with *Xho*I and *Sal*I sites for the left arm or *Sal*I and *Cla*I sites for the right arm, respectively. Following the digestion of the 2.0-kb fragment with *Xho*I and *Sal*I, and the 4.0-kb fragment with *Sal*I and *Cla*I, each fragment was ligated into each site of pBluescript. A *Sal*I restriction site was engineered in place of the start codon in exon 1. The *FRT-PGK-gb2-Neo-FRT-Stop* cassette was cloned into the *SalI* site inserted into the targeting vector. Finally, the targeting construct was abutted to a MC1-DTa negative-selection cassette and linearized. The linearized targeting construct was introduced by electroporation into C57BL/6-derived JN/2 recombinant embryonic stem cell (ESC) and neomycin-resistant clones were first screened for homologous recombination by PCR utilizing a pair of the following oligonucleotides: Primer 1 (5′-ATA TGT AGT GTC TGG TCA GGA TAA TAG TTG-3′) and Primer 2 (5′-ATA ACC TCC TCT CCT ATG GTA CCT AAA C-3′). *Spe*I-digested genomic DNA of positive clones was then screened by Southern blotting with a 3′ external single-copy probe corresponding to a 0.607-kb fragment, which was amplified by PCR using these primers: 5′-TAT ACA CAC CTA TGA ATG CAT GCT C-3′ and 5′-CTC TGA CTA AAC CCC ATC TTG ATA A-3′. When tested on *Spe*I-digested DNA, it hybridized either to a 13.3-kb WT fragment or to a 10.7-kb recombinant fragment. ESC clones bearing the correctly targeted locus were injected into BALB/c blastocysts, and chimeric male offspring were mated with female C57BL/6 mice to obtain heterozygotes, which were then crossed to obtain homozygotes. Transmission of the targeted allele was confirmed by PCR with Primer 1 and Primer 3 (5′-CTT TAT ATT TCA TTT TTG CTC AGG CTT C-3′). The mutant mice were cross-mated for more than nine generations with B6.FLIP mice to excise the flanked FRT sites by Flp-recombinase, and 8- to 12-week-old *Cd103*^+/+^ littermates were used as WT mice. Then, *Cd103*^+/−^ littermates were crossed to obtain homozygotes, and transmission of the targeted allele was confirmed by PCR with Primer 1 and Primer 3.

### Tissues and cell isolation

Isolations of tissue and cells were performed according to the precious reports^4,11,16^. To prepare single-cell suspensions from Spl or PLNs were digested with 400 U/ml collagenase type III (Worthington Biochemical) at 37 °C for 20 min or 30 min and were ground between glass slides. Cell suspensions of Spl were further treated with RBC lysis buffer (Sigma-Aldrich). Ear skin fragments were dissected and digested with collagenase type III at 60 min as described above. Single-cell suspensions were obtained by forcing through a 100-μm cell strainer (BD Biosciences). CD11c^+^ DCs were purified by AutoMACS with mouse CD11c (N418) Microbeads. CD3^+^CD8^+^ T cells were purified from splenocytes with mouse CD8 T-lymphocyte Enrichment Set-DM (BD Biosciences).

### Flow cytometry

Flow cytometry were performed according to the precious reports^4,11,16^. Cells were stained with fluorescein-conjugated mAbs and Ab to mouse, CD4 (RM4–5), CD8α (53–6.7), CD11c (HL3), CD40 (3/23), CD45R/B220 (RA3–6B2), CD45.2 (104), CD80 (16–10A1), CD86 (GL1), CD103 (M290), I-A/I-E (M5/114.15.2), Gr-1 (RB6–8C5), IL-17A (TC11–18H10), isotype-matched control mAb (cont. Ig) (BD Biosciences), CD3ε (145–2C11), CD11b (M1/70), B7-DC (TY25), γδTCR (GL3), Foxp3 (FJK-16s), and IL-22 (IL22JOP) (eBiosciences), CD90.2 (53–2.1), F4/80 (BM8), Siglec-H (551) (BioLegend). For the intracellular expression of cytokines, cells were incubated for 4 hrs with phorbol 12-myristate 13-acetate (PMA, 50 ng/ml; Sigma-Aldrich) and ionomycin (500 ng/ml; Sigma-Aldrich) plus Brefeldin A (eBiosciences) during the final 2 hrs. Subsequently, the cells were resuspended in Fixation-Permeabilization solution (eBiosciences) and intracellular cytokine staining was carried out according to the manufacturer’s directions. Fluorescence staining was analyzed with a FACSVerse flow cytometer and FlowJo software (both from BD Biosciences).

### Quantitative reverse transcriptase polymerase chain reaction (qRT-PCR)

qPCR was performed according to the precious reports^11,16^. Total RNA from ear skin was extracted with ISOGEN II (NIPPON GENE) and the first-strand complementary DNA (cDNA) was synthesized from 1 μg of total RNA using the PrimeScript RT Master Mix (Takara) according to the manufacturer’s instructions. Transcriptional expression levels were analyzed by using SYBR^®^ Premix Ex Taq II on Thermal Cycler Dice (Takara) with specific primer pairs (Supplementary Table 1) after normalization for the expression of *Gapdh*.

### Skin inflammation

Induction and analysis of IMQ-induced psoriasiform skin inflammation were performed according to the precious reports^11,16^. Mice were treated topically with either 25 mg of 5% IMQ cream (Mochida Pharmaceutical) or petrolatum (Wako Pure Chemicals) as a control on the left ear every day for 6 days. The severity of the ear skin inflammation of each mouse was determined daily by ear thickness using digital calipers (PK-1012CPX; Mitsutoyo), and ear photographs were taken at days 0 and 6 after topical application of IMQ cream on the ear skin.

### Histopathologic assessment

Assessment of the Histopathology was performed according to the precious reports^11,16^. Ear tissues were fixed with 4% paraformaldehyde (PFA) in PBS and embedded in paraffin. The tissue sections (5 μm thickness) were stained with H&E. The stained slides were examined with a bright-field microscope (BX53; Olympus). The thickness of ear epidermis and dermis was measured for thickness using ImageJ (National Institutes of Health) by a blinded observer.

### Immunohistochemical analysis

Immunohistochemical analysis was performed according to the precious reports^11,16^. For cutaneous detection of Gr-1^+^ granulocytes^11,16^, the ear skin was embedded in OCT compound (Sakura Fineteck) and frozen in liquid N_2_. The tissue block was sectioned with a cryostat at 5–7 μm. Frozen sections were fixed with cold acetone and blocked in PBS containing 5% normal rat serum. Subsequently, slide was stained with FITC-conjugated anti-Gr-1 mAb (BD Biosciences) and mounted with Vectashield (Vector laboratories). The stained slides were analyzed with a All-in-One Fluorescence Microscope (BZX-710; KEYENCE).

### Culture of DCs

According to the previous reports^11,16^, DCs (10^5^) were cultured with or without IMQ (10 μg/ml; Invivogen) for 5 hrs in 96-well flat-bottomed plates (BD Bioscience).

### *In vitro* T_C_17 cell differentiation assay

As described in previous reports with some modification^4,11,16^, naïve CD8^+^ T cells (5 × 10^4^) were cultured with cDCs (2.5 × 10^4^) in combination with anti-CD3ε mAb (1 μg/ml; 145-2C11, Biolegend), anti-IFN-γ mAb (5 μg/ml; R4-6A2, BD Biosciences), recombinant human transforming growth factor (TGF)-β (2 ng/ml; Wako Pure Chemicals) and IL-6 (40 ng/ml) for 5 days in 96-well round-bottomed plates (BD Bioscience). In another experiment, naïve CD8^+^ T cells (4 × 10^4^) were cultured in combination with anti-CD3ε mAb (1 μg/ml), anti-CD28 mAb (1 μg/ml), anti-IFN-γ mAb (5 μg/ml), recombinant human TGF-β1 (2 ng/ml) and IL-6 (20 ng/ml) for 5 days in 96-well flat-bottomed plate. Analysis of IL-17A and IFN-γ expression among gated CD8^+^ T cells was performed by flow cytometry as described above.

### Statistical analysis

Statistical analysis was performed as described previously^4,11,16^. Data are the means ± s.d. from three to eight individual samples in a single experiment, and we performed at least four independent experiments. The statistical significance of differences between groups was analyzed using Student’s *t*-test (two-tailed). P values <0.05 or 0.01 were considered statistically significant.

## Supplementary information

Supplementary Information.
