# Author Correction: Pivotal role of CD103 in the development of psoriasiform dermatitis

**DOI:** 10.1038/s41598-020-71156-x

**Published:** 2020-09-29

**Authors:** Takehito Fukui, Tomohiro Fukaya, Tomofumi Uto, Hideaki Takagi, Junta Nasu, Noriaki Miyanaga, Yotaro Nishikawa, Haruhiko Koseki, Narantsog Choijookhuu, Yoshitaka Hishikawa, Yoshihiro Yamashita, Katsuaki Sato

**Affiliations:** 1 Division of Immunology, Department of Infectious Diseases, Faculty of Medicine, University of Miyazaki, 5200 Kihara, Kiyotake, Miyazaki 889-1692 Japan; 2 Department of Oral and Maxillofacial Surgery, Faculty of Medicine, University of Miyazaki, 5200 Kihara, Kiyotake, Miyazaki 889-1692 Japan; 3 Department of Otolaryngology, Head and Neck Surgery, Faculty of Medicine, University of Miyazaki, 5200 Kihara, Kiyotake, Miyazaki 889-1692 Japan; 4 Department of Dermatology, Faculty of Medicine, University of Miyazaki, 5200 Kihara, Kiyotake, Miyazaki 889-1692 Japan; 5 Laboratory for Developmental Genetics, RIKEN Center for Integrative Medical Sciences, 1-7-22 Suehiro-cho, Tsurumi-ku, Yokohama, Kanagawa 230-0045 Japan; 6 Division of Histochemistry and Cell Biology, Department of Anatomy, Faculty of Medicine, University of Miyazaki, Miyazaki, 889-1692 Japan; 7 Japan Agency for Medical Research and Development (AMED), 1-7-1 Otemachi, Chiyoda-ku, Tokyo, 100-0004 Japan

Correction to: *Scientific Reports* 10.1038/s41598-020-65355-9, published online 20 May 2020

This Article contains errors in the Methods section, under the subheading ‘Generation of *Cd103*^−/−^ mice’,

“The linearized targeting construct was introduced by electroporation into C57BL/6-derived JN/2 recombinant embryonic stem cell (ESC) and neomycin-resistant clones were first screened for homologous recombination by PCR utilizing a pair of the following oligonucleotides: Primer 1 (5′-ATA TGT AGT GTC TGG TCA GGA TAA TAG TTG-3′) and Primer 2 (5′-ATA ACC TCC TCT CCT ATG GTA CCT AAA C-3′).”

should read:

“The linearized targeting construct was introduced by electroporation into C57BL/6-derived JN/2 recombinant embryonic stem cell (ESC) and neomycin-resistant clones were first screened for homologous recombination by PCR utilizing a pair of the following oligonucleotides: Primer 1 (5′-ATA TGT AGT GTC TGG TCA GGA TAA TAG TTG-3′) and Primer 3 (5′-ATA ACC TCC TCT CCT ATG GTA CCT AAA C-3′).”

“Transmission of the targeted allele was confirmed by PCR with Primer 1 and Primer 3 (5′-CTT TAT ATT TCA TTT TTG CTC AGG CTT C-3′). The mutant mice were cross-mated for more than nine generations with B6.FLIP mice to excise the flanked FRT sites by Flp-recombinase, and 8- to 12-week-old *Cd103*^+/+^ littermates were used as WT mice. Then, *Cd103*^+/−^  littermates were crossed to obtain homozygotes, and transmission of the targeted allele was confirmed by PCR with Primer 1 and Primer 3.”

should read:

“Transmission of the targeted allele was confirmed by PCR with Primer 1 and Primer 2 (5′-CTT TAT ATT TCA TTT TTG CTC AGG CTT C-3′). The mutant mice were cross-mated for more than nine generations with B6.FLIP mice to excise the flanked FRT sites by Flp-recombinase, and 8- to 12-week-old *Cd103*^+/+^ littermates were used as WT mice. Then, *Cd103*^+/−^  littermates were crossed to obtain homozygotes, and transmission of the targeted allele was confirmed by PCR with Primer 1 and Primer 2.”

