# Supplementary material for: Pivotal role of CD103 in the development of psoriasiform dermatitis
Source: Sci Rep. 2020 May 20;10:8371. doi: 10.1038/s41598-020-65355-9 (PMC7239860; doi:10.1038/s41598-020-65355-9)
Supplement: Supplementary file 1 — Supplementary Information. [file 41598_2020_65355_MOESM1_ESM.pdf]

Supplementary Information

Pivotal role of CD103 in the development of psoriasiform dermatitis

Takehito Fukui[1,2], Tomohiro Fukaya[1,7], Tomofumi Uto[1,7], Hideaki Takagi[1,7], Junta Nasu[1,2], Noriaki Miyanaga[1,3], Yotaro Nishikawa[1,4], Haruhiko Koseki[5], Narantsog Choijookhuu[6], Yoshitaka Hishikawa[6], Yoshihiro Yamashita[2] & Katsuaki Sato[1,7]

[1]Division of Immunology, Department of Infectious Diseases, Faculty of Medicine, University of Miyazaki, 5200 Kihara, Kiyotake, Miyazaki 889-1692, Japan

[2]Department of Oral and Maxillofacial Surgery, Faculty of Medicine, University of Miyazaki, 5200 Kihara, Kiyotake, Miyazaki 889-1692, Japan

[3]Department of Otolaryngology, Head and Neck Surgery, Faculty of Medicine, University of Miyazaki, 5200 Kihara, Kiyotake, Miyazaki 889-1692, Japan

[4]Department of Dermatology, Faculty of Medicine, University of Miyazaki, 5200 Kihara, Kiyotake, Miyazaki 889-1692, Japan

[5]Laboratory for Developmental Genetics, RIKEN Center for Integrative Medical Sciences, 1-7-22 Suehiro-cho, Tsurumi-ku, Yokohama, Kanagawa 230-0045, Japan

[6]Division of Histochemistry and Cell Biology, Department of Anatomy, Faculty of Medicine, University of Miyazaki, Miyazaki 889-1692, Japan

[7]Japan Agency for Medical Research and Development (AMED), 1-7-1 Otemachi, Chiyoda-ku, Tokyo 100-0004, Japan

Correspondence should be addressed to: Katsuaki Sato, Division of Immunology, Department of Infectious Diseases, Faculty of Medicine, University of Miyazaki, 5200 Kihara, Kiyotake, Miyazaki 889-1692, Japan

Phone: +81-985-85-9815, Fax: +81-985-85-9899,

e-mail: katsuaki_sato@med.miyazaki-u.ac.jp

Supplementary Table 1

RT-PCR primer	Sequence
Gapdh	F-5’-AAATTCAACGGCACAGTCAAG-3’
	R-5’-TGGTGGTGAAGACACCAGTAG-3’
Il1a	F-5’-TCCAGGGCAGAGAGGGAGT-3’
	R-5’-GGAACTTTGGCCATCTTGATTT-3’
Il1b	F-5’-GAAGAAGAGCCCATCCTCTG-3’
	R-5’-TCATCTCGGAGCCTGTAGTG-3’
Il6	F-5’-GACTGATGCTGGTGACAACC-3’
	R-5’-CCTCCGACTTGTGAAGTGG-3’
Il10	F-5’-TGCAGCAGCTCAGAGGGTT-3’
	R-5’-TGGCCACAGTTTTCAGGGAT-3’
Il12a	F-5’-AAACCAGACCCGCCCAAGAAC-3’
	R-5’-AAAAAGCCAACCAAGCAGAAGACAG-3’
Il17a	F-5’-CTGCTGAGCCTGGCGGCTAC-3’
	R-5’-CATTGCGGTGGAGAGTCCAGGG-3’
Il19	F-5’-GCCAACTCTTTCCTCTGCGT-3’
	R-5’-GGTGGCTTCCTGACTGCAGT-3’
Il20	F-5’-GACCCCTGACCACCATACCC-3’
	R-5’-CCATTGCTTCTTCCCCACAA-3’
Il22	F-5’-CAGCTCCTGTCACATCAGCGGT-3’
	R-5’-AGGTCCAGTTCCCCAATCGCCT-3’
Il23a	F-5’-TCCGTTCCAAGATCCTTCG-3’
	R-5’-GAACCTGGGCATCCTTAAGC-3’
Tnf	F-5’-GCCCACGTCGTAGCAAACCAC -3’
	R-5’-GCAGGGGCTCTTGACGGCAG -3’
Cxcl1	F-5’-GCCTATCGCCAATGAGCTG-3’
	R-5’-ATTCTTGAGTGTGGCTATGA-3’
Cxcl2	F-5’-ATGCCTGAAGACCCTGCCAAG-3’
	R-5’-GGTCAGTTAGCCTTGCCTTTG-3’
S100a7	F-5’-GGGCAGCTGACAAAAACAAG-3’
	R-5’-TGGAACTGGAGATGGTAGTCC-3’
S100a8	F-5’-CCATGCCCTCTACAAGAATG-3’
	R-5’-ATCACCATCGCAAGGAACTC-3’

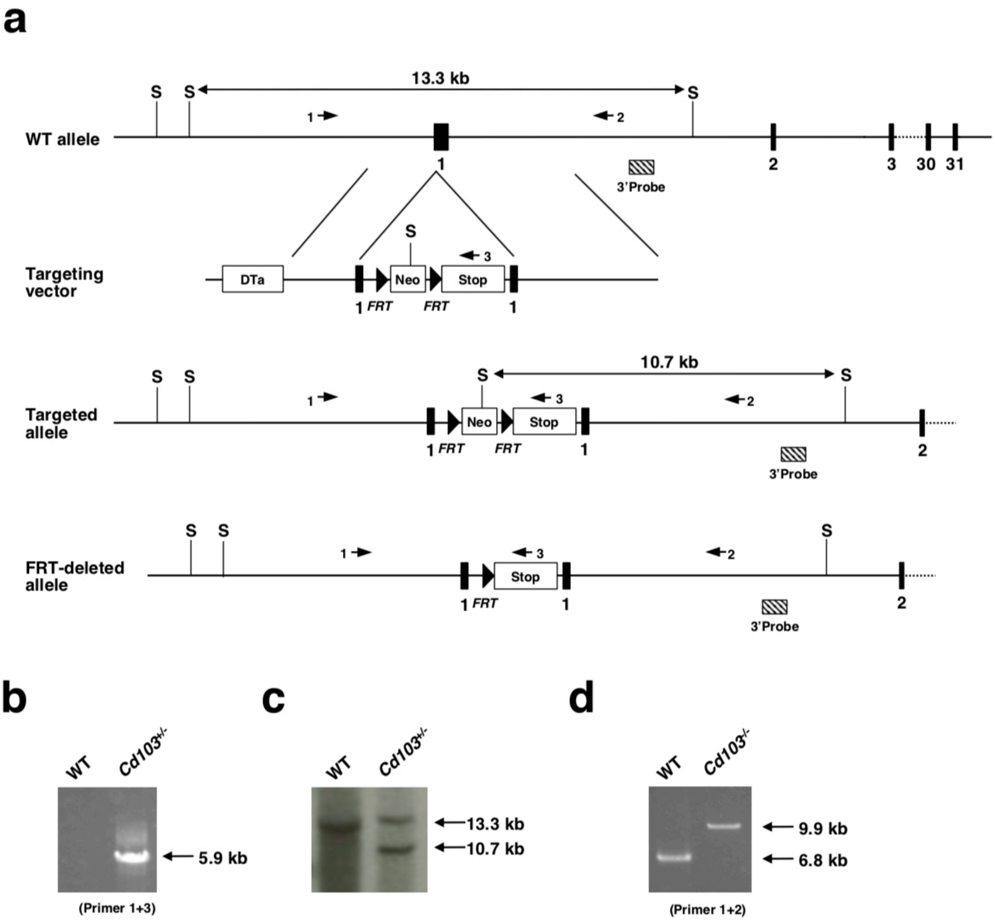

Supplementary Figure 1. Generation and identification of *Cd103*$^{-/-}$ mice. (**a**) Strategy used to produce the *Cd103*$^{-/-}$ mice. (1) Partial restriction map of the WT *Cd103* gene. Exons are depicted as black boxes. The restriction site indicated is S: *Spe*I (2) Targeting vector used to introduce the mutations in the *Cd103* gene. A *Sal*I site engineered in place of the start codon in exon 1 of the *Cd103* gene was used to clone the *FRT-PGK-gb2-Neo-FRT-Stop* cassette. DTa: diphtheria toxin a expression cassette, Stop: Stop cassette, Neo: Neo cassette. The Neo cassette is shown bracketed by FRT sites (filled triangles). (3) Structure of the targeted allele following homologous ESC clones. (4) Structure of the *Cd103* allele following expression of FLP recombinase and excision of the Neocassette in mutant mice. The 3' external single-copy probe (a hatched box) and the PCR primers at the 5' end (blackarrows) used to verify proper homologous recombination events are shown. **(b,c)** DNA-PCR **(b)** and Southern blot **(c)** analysis of WT and recombinant ESC clones. **(d)** Genotyping of tail DNA from WT mice and homozygous mice for the *Cd103* allele by DNA-PCR. The results are representative of at least three independent experiments.

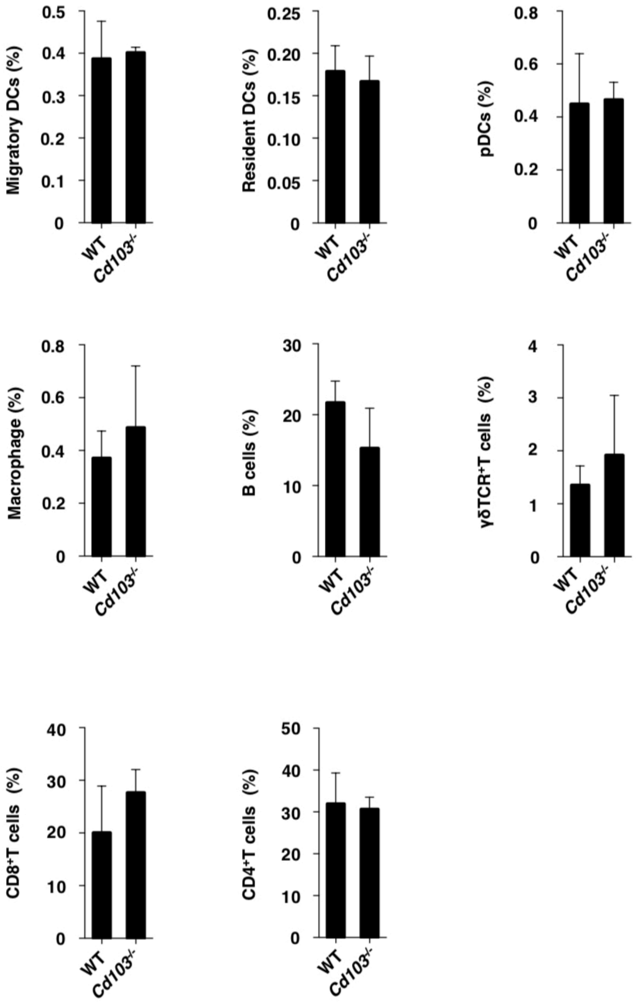

Supplementary Figure 2. Constituencies of leukocytes in PLNs in *Cd103*$^{-/-}$ mice. The frequency of the indicated leukocytes in skin-draining PLNs was analyzed by flow cytometry in WT mice (n=3) and *Cd103*$^{-/-}$ mice (n=3). Data are the mean ± s.d. from three individual samples in a single experiment. The results are representative of at least three independent experiments.

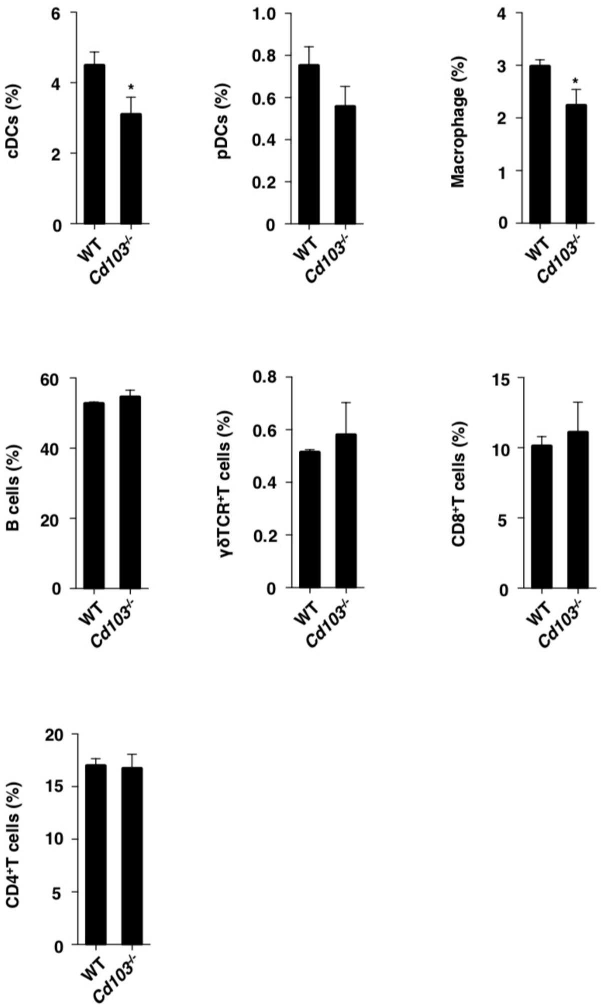

Supplementary Figure 3. Constituencies of splenic leukocytes in *Cd103*$^{-/-}$ mice. The frequency of the indicated leukocytes in Spl was analyzed by flow cytometry in WT mice (n=3) and *Cd103*$^{-/-}$ mice (n=3). Data are the mean ± s.d. from three individual samples in a single experiment. **P* < .05 compared with WT mice. The results are representative of at least three independent experiments.

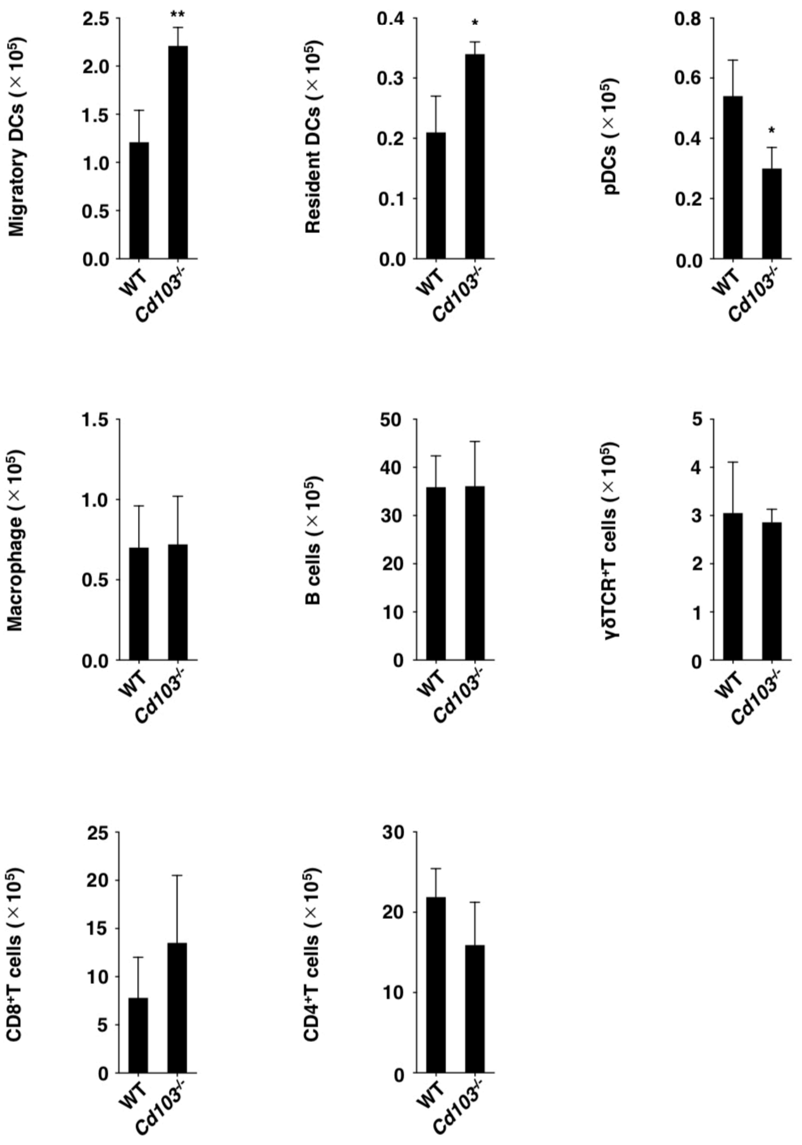

Supplementary Figure 4. Absence of CD103 enhances the accumulation of inflammatory leukocytes in skin-draining PLNs. The absolute cell numbers of leukocytes in the skin-draining PLNs at day 6 after topical application of IMQ on the left ear skin every day for 6 days in WT mice (n=5) and *Cd103*$^{-/-}$ mice (n=5). Data are the mean ± s.d. in three to four individual samples in a single experiment. *$P < .05$,**$P < .01$ compared with WT mice. The results are representative of at least three independent experiments.

a

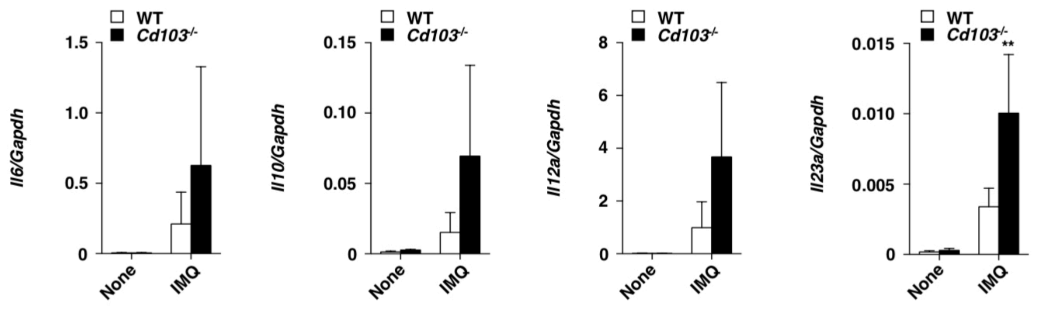

b

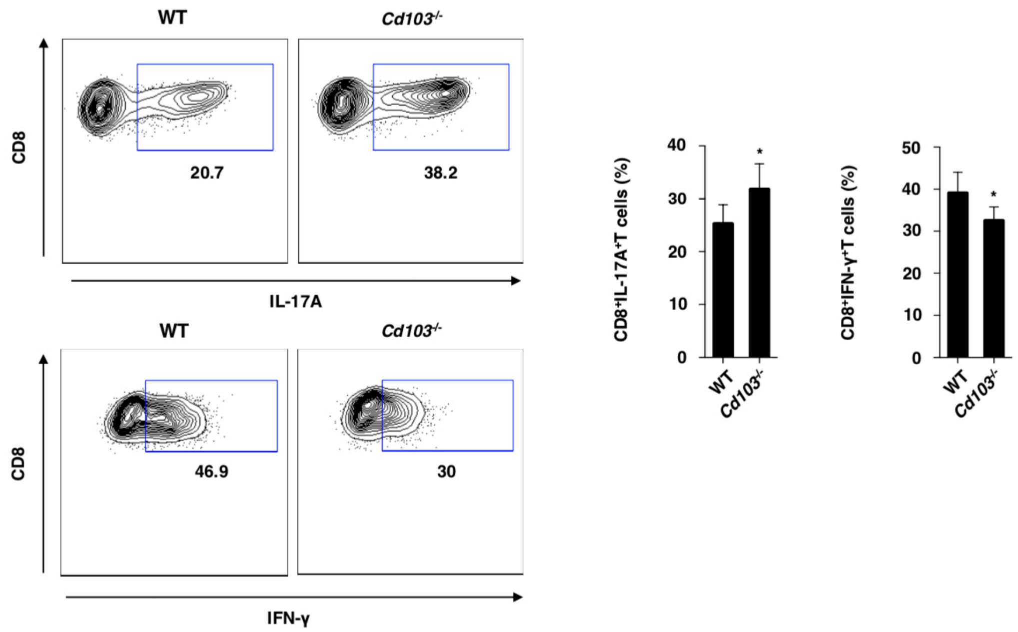

Supplementary Figure 5. CD103 deficiency enhances the TLR7-mediated activation of cDCs. (**a**) Splenic cDCs derived from WT mice and *Cd103*$^{-/-}$ mice were not stimulated (None) or stimulated with IMQ (IMQ). The transcriptional expressions of cytokines in cDCs. Data are the mean ± s.d. from three to six individual samples in a single experiment. ***P* < .01 compared with WT mice. (**b**) CD8^{+} T cells were cultured with cDCs obtained from the skin-draining PLNs in WT mice and *Cd103*$^{-/-}$ mice for 5 days under T_c17-polarized culture conditions. The frequency of IL-17A- and IFN-γ-producing cells among CD8^{+} T cells. Data are presented as a contour plot, and numbers mean the proportion of the indicated cell populations in each gate (left panel). Data are the mean ± s.d. in three individual samples in a single experiment (right panel). **P* < .05 compared with WT mice. The results are representative of at least three independent experiments.

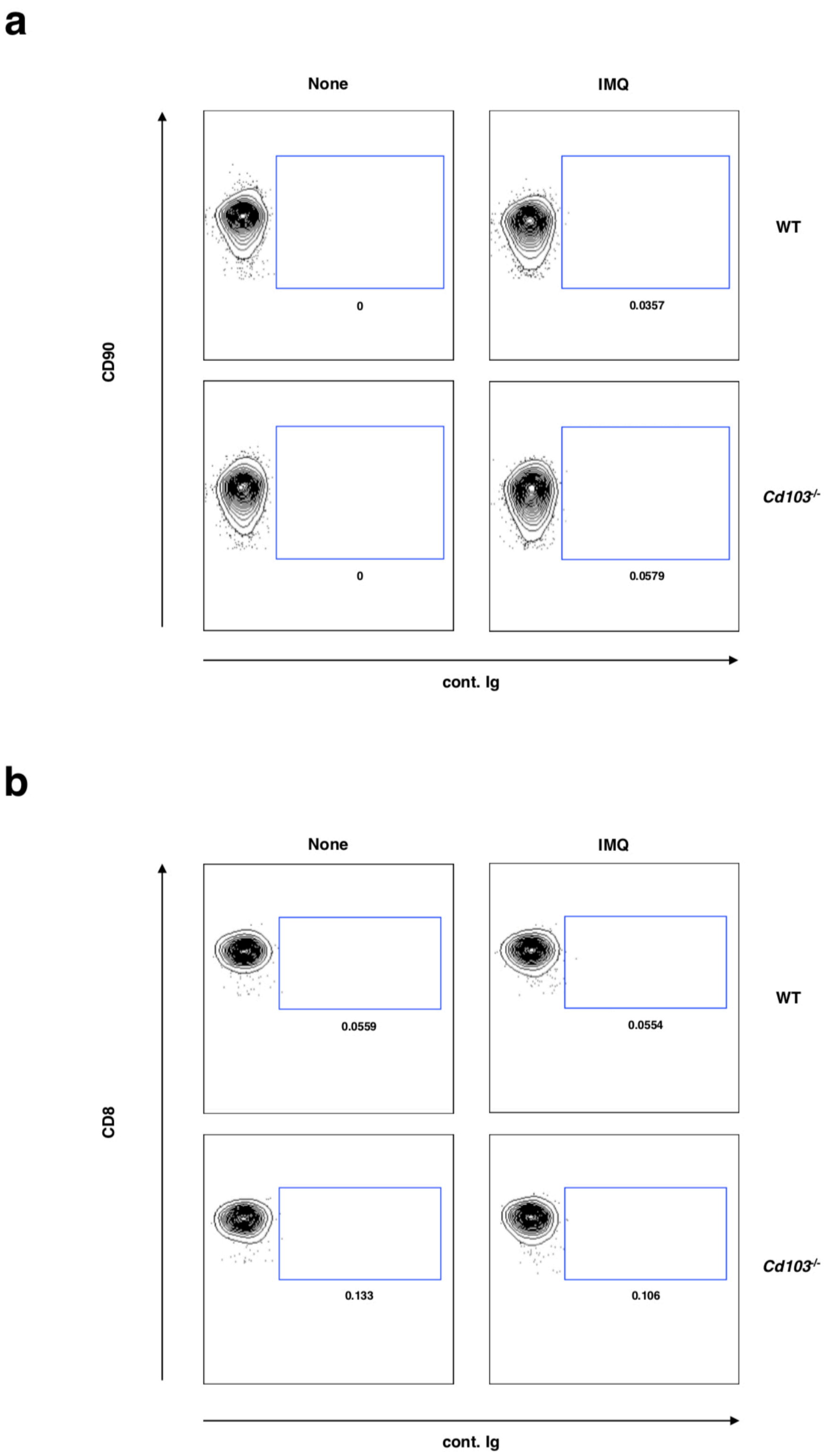

Supplementary Figure 6. Staining of IL-17A-prodcing lymphocytes with isotype control mAb. WT mice (n=3) and *Cd103*$^{-/-}$ mice (n=3) were treated topically with IMQ on the left ear skin every day for 6 days. Data are presented as a contour plot for isotype control staining (cont. Ig) of innate lymphocytes (**a**) and CD8^{+} T cells (**b**) in the skin-

draining PLNs. The results are representative of at least three independent experiments.

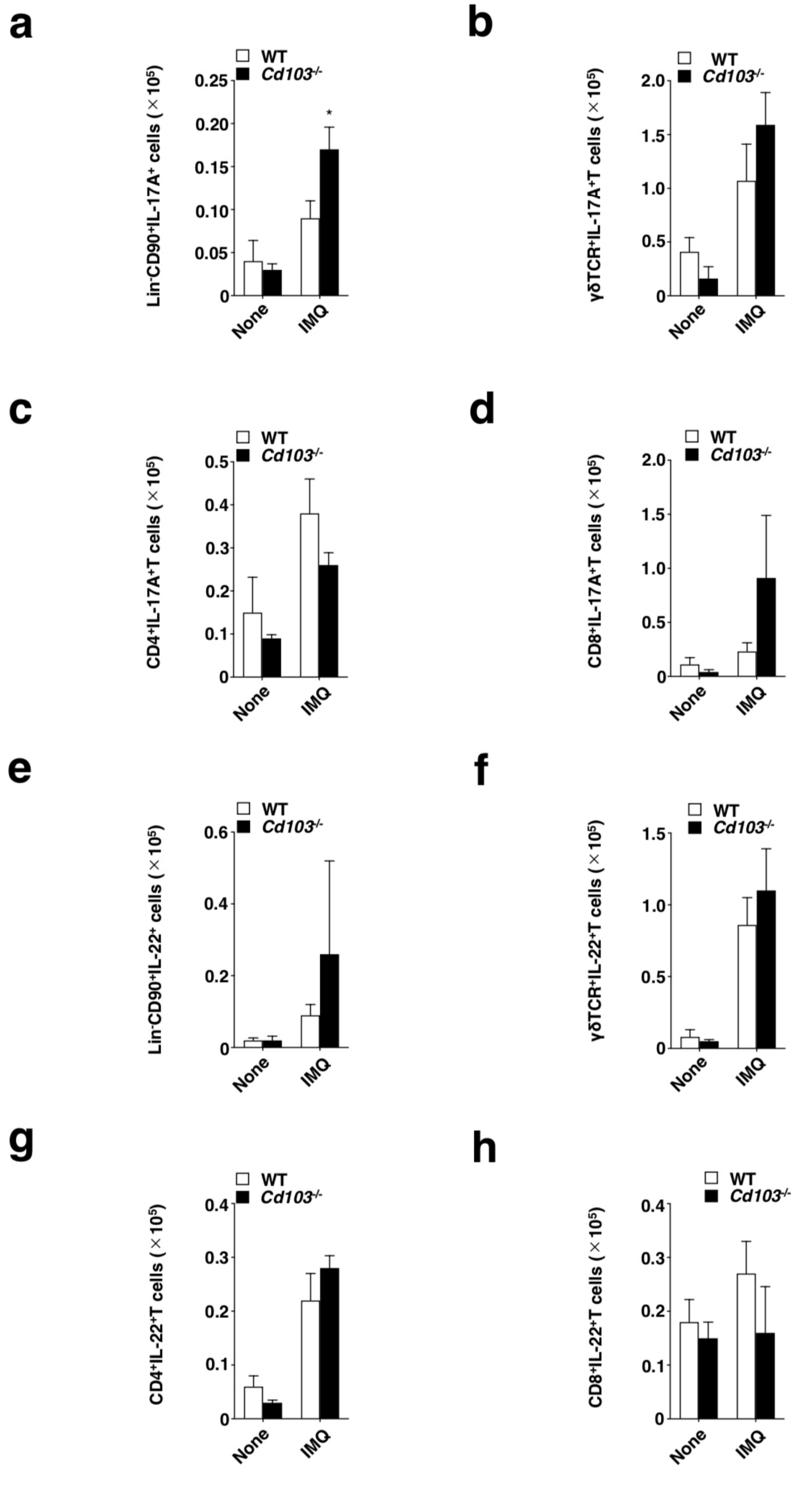

Supplementary Figure 7. CD103 deficiency enhances the generation of IL-17A-prodcing lymphocytes in the skin-draining PLNs in the progression of psoriasiform skin

inflammation. WT mice (n=3) and *Cd103*$^{-/-}$ mice (n=3) were treated topically with IMQ on the left ear skin every day for 6 days. The absolute cell numbers of IL-17A-producing cells (**a-d**) and IL-22-producing cells (**e-h**) among innate lymphocytes (**a,e**), γδTCR^{+} T cells (**b,f**), CD4^{+} T cells (**c,g**), and CD8^{+} T cells (**d,h**) in the skin-draining PLNs at days 0 and 6. Data are the mean ± s.d. in three individual samples in a single experiment. *P < .05, **P < .01compared with WT mice. The results are representative of at least three independent experiments.

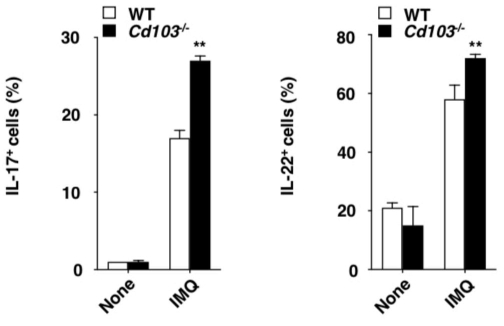

Supplementary Figure 8. CD103 deficiency promotes the generation of IL-17A-prodcing lymphocytes in the dermal tissues in the development of psoriasiform skin inflammation. WT mice (n=3) and *Cd103*$^{-/-}$ mice (n=3) were treated topically with IMQ on the left ear skin every day for 6 days. The frequencies of IL-17A-producing cells and IL-22-producing cells CD45.2^{+} leukocytes in the dermal tissues at days 0 and 6. Data are the mean ± s.d. in three individual samples in a single experiment. *P < .05, **P < .01compared with WT mice. The results are representative of at least three independent experiments.

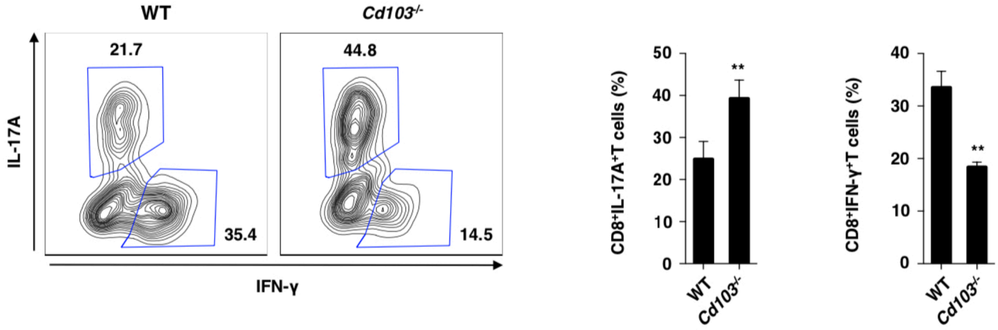

Supplementary Figure 9. Influence of CD103 deficiency on the generation of T_c17 cells. $CD8^+$ T cells derived from WT mice and *Cd103*$^{-/-}$ mice were cultured for 5 days under T_c17-polarized culture conditions. The frequency of IL-17A- and IFN-γ-producing cells among $CD8^+$ T cells. Data are presented as a contour plot, and numbers mean the proportion of the indicated cell populations in each gate (left panel). Data are the mean ± s.d. in three to five individual samples in a single experiment (right panel). $^{**}P < .01$ compared with WT mice. The results are representative of at least three independent experiments.

Figure S1b

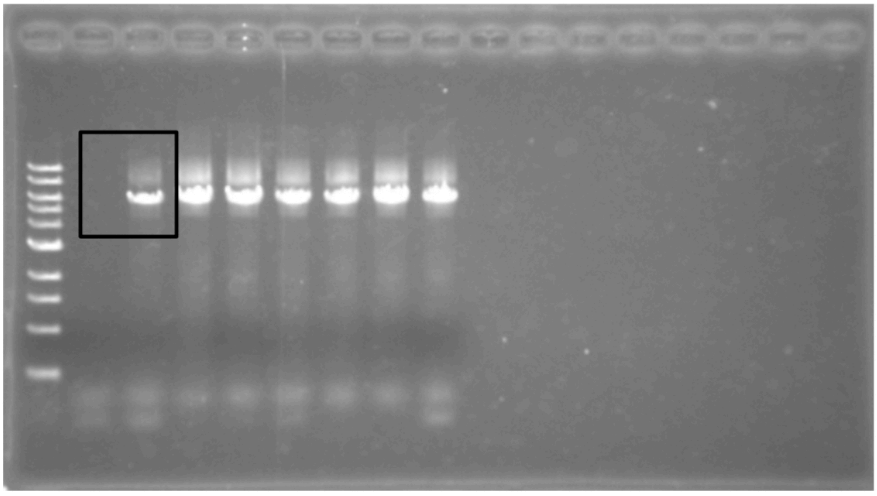

Figure S1c

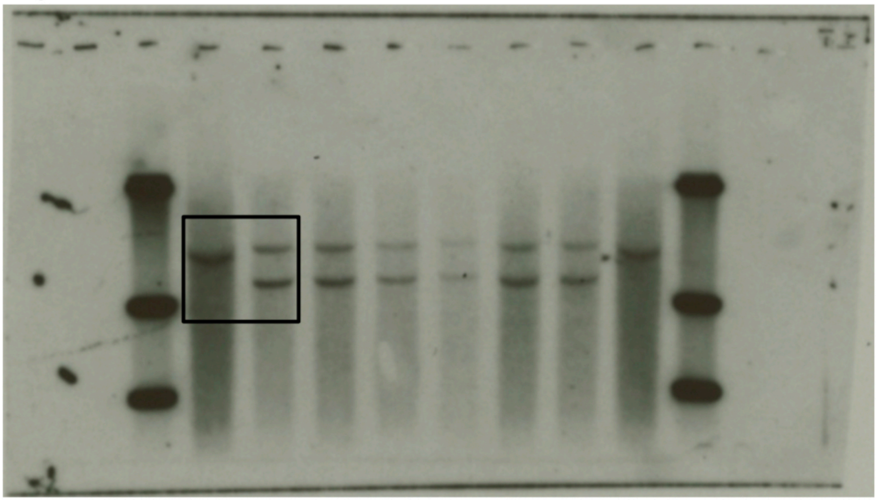

Figure S1d

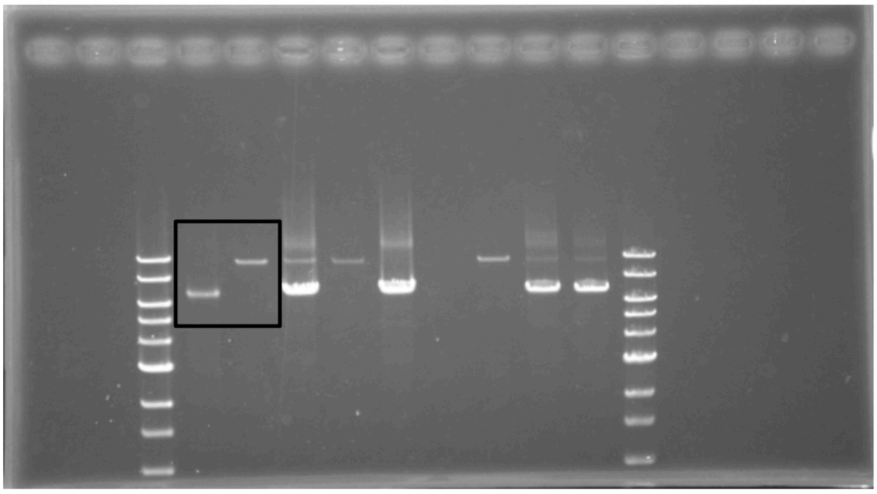

Supplementary Figure 10. Full-length gels and blots of Supplementary Figure 1b-d.
